# The evolution of birth-order-specific son preference and compulsory primary education: Evidence from Vietnam

**DOI:** 10.1371/journal.pone.0335527

**Published:** 2025-12-01

**Authors:** Ryo Hasegawa

**Affiliations:** Cornell University, Ithaca, New York, United States of America; Institut National dÉtudes Demograpiques: INED, FRANCE

## Abstract

This paper examines birth-order-specific son preference and the effect of compulsory primary education on the male-skewed preference. Using four waves of Vietnamese decennial censuses from 1989 to 2019, the study finds that a desire to have a son at the first birth order—referred to as son-starting behavior—has emerged over time, particularly among small families. Exploiting a disproportionate increase in education in ethnic minorities triggered by the compulsory primary education law in 1991, difference-in-differences estimates suggest that female primary education reduces son-starting behavior by 3.13 percentage points. While this does not fully lead to gender neutrality across all birth orders, the magnitude is economically meaningful to mitigate the skewed preference for the first-born child. Mechanism analysis suggests that the effects could be mediated through an increased likelihood of marrying more educated husbands.

## 1 Introduction

Son preference, a desire to have at least one son, is prevalent in many societies, but particularly in East and South Asia countries [1,2]. It manifests most directly in the form of a higher sex ratio at birth [1]. The consequences of son preference range from sex-selective abortions [3], infanticides [4], short birth-spacing [5,6] and less breastfeeding for girls [7]. The results are a widened gender gap in health [8] and education [9], a shortage of women in the marriage market [10] and a higher crime rate [11]. Given these negative consequences of son preference, finding policies to address it is of great importance, but research on this topic is scarce. This paper explores how compulsory primary education affects the birth-order-specific son preference in Vietnam, a country that exhibits the fifth highest sex ratio at birth in the world [12].

I first present descriptive evidence of the evolution of son preference by highlighting the role of birth-order-specific sex-ratio. Then, leveraging the disproportionate increase in women’s education among ethnic minorities triggered by the 1991 compulsory primary education law, I employ a difference-in-differences framework to demonstrate that female education has the potential to reduce son-starting behavior. The results are robust, supported by a battery of robustness checks that indicate the parallel trends assumption is not violated.

This paper contributes to the literature on the nature of son preference in two ways. First, it reveals that the manifestation of son preference can vary significantly depending on birth order. This birth-order-specific preference has not been considered in the previous literature. This paper squares with this by exploring Vietnam where the “son-starting” behavior—defined as a desire to have a son as the first child—became more pronounced over years. Second, to my knowledge, this is the first paper to explore the causal relationship between son-starting behavior and female education.

## 2 Data and stylized facts about birth order and sex composition

I start by examining the evolution of son preference, focusing on birth order and sibling size. The objective of this exercise is to illustrate how son preference manifests differently at each birth order conditional on the sibling size. I use the Vietnam Population and Housing Censuses data from 1989, 1999, 2009, and 2019. The data are the 5%, 3%, 15%, and 8.5% nationally representative samples of the population [13].

The major challenge in accurately identifying the birth order of children within a household is that the Census neither collects the information of the birth order of children nor records the complete birth history of women. Additionally, as children get older, they might leave their parents but this attrition is not observable from the Census. To overcome these challenges, first, I identify a biological linkage between children and their mothers. Then, I use child’s birth year and birth month to construct the birth order variable. To verify accuracy, I compare the total number of ordered siblings with the number of children whose mothers have ever had in the past. If there are discrepancies, I drop the samples. While this process necessarily excludes children whose mothers are not in the same household and had deceased children, it leaves more samples with more accuracy than the alternative where I relied on the relation to the household head. Since I use the biological linkage, the adopted children are removed at this stage.

To focus on households that have completed fertility, I further restrict the sample to households in which women are 35 years old or older, as households with women of that age are likely to have completed fertility. Households with twins and triplets are removed as their birth orders cannot be determined. S1 Table, S2 Table, S3 Table and S4 Table report the details of how the sample composition changes at each step by survey year. Notably, the mean gender composition and other observable characteristics remain not greatly changed across all survey years.

**Fig 1:**
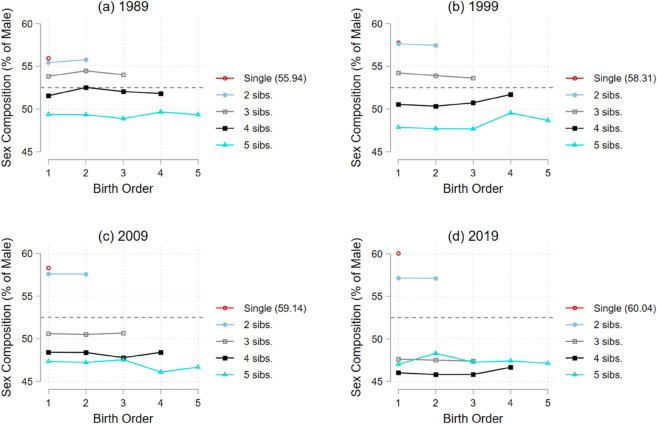
Sex Composition and birth order from 1989-2019. Notes: Fig 1 shows a simple descriptive statistics about the sex composition of siblings given the total number of siblings. The gender-neutral state is represented by $52.5=50\;\times \;\frac{105}{100}$ The statistics are weighted by sampling weights.

Fig 1 illustrates the relationship between birth order and sex composition, conditional on household size. Two distinct patterns emerge. First, the curve for households with fewer children lies above that for larger households, indicating that smaller families tend to have a higher proportion of male firstborns. This pattern aligns with findings from Jayachandran (2017) [14], who shows that the desired sex ratio tends to rise as fertility declines. Second, and more strikingly, among women aged 35 or older in 1989, the sex composition at the first birth order was relatively stable across different household sizes during this earlier period. After 1999, however, this pattern diverges: while lower sex ratios persist among families with three or more children, a disproportionate number of smaller families (with one or two children) began childbearing with sons. I refer to this shift as “son-starting”—a fertility behavior in which households exhibit a preference for having a son as their first child. In a low-fertility regime, son-starting behavior is expected to rise, as families have fewer opportunities to continue childbearing if their preferred sex is not achieved early. I replicate this analysis using alternative maternal age thresholds (age 40 in S1 Fig and age 45 in S2 Fig) and by ethnicity (ethnic majority in S3 Fig and ethnic minorities in S4 Fig). The son-starting pattern is consistently observed across these subsamples.

## 3 Birth-order-specific preference and female education

As shown in the previous section, son-starting behavior is particularly pronounced among smaller households. However, its underlying determinants remain unclear. To explore this, I use the 2019 Census and construct an analytical sample consisting of women with accurately determined birth order information, following the procedure outlined in the previous section, and supplement it with women who have no children. Using this sample, I estimate regression models to assess whether the expansion of compulsory education mitigated son-starting behavior.

### 3.1 Compulsory primary education

The compulsory primary education law was implemented in 1991. Prior to its enactment, school attendance was not mandatory for all children. This law (No.56-LCT/HDNN8), effective from August 1991, mandates that all children aged 14 or younger complete five years of primary education (Article 1) and that all children start grade 1 at age 6, except in special cases such as children with health problems and difficulties in attending schools (Article 8 & Article 10). Consequently, children are expected to finish primary education by age 11. Considering that delays in starting school or repeating grades are common (S5 Fig), I use 1977 as the cutoff year and define the cohort born between 1972 and 1976 as unaffected and the cohort born between 1977 and 1985 as affected. The 1985 cohort is chosen because it was the first to be fully exposed to the education reform.

In addition to birth timing, I use ethnicity as a second source of variation. A major empirical challenge in considering location aspects to study the relationship between education and fertility is migration: neither birthplace nor current residence might not capture relevant exposure to the education reform as people move from one place to another and the information is often not available. This complicates the use of geographic variation to measure treatment effects and requires additional assumptions or sample restrictions [15]. To address this limitation, particularly when policy implementation and relevant outcomes are temporally distant, I use ethnicity as a time-invariant proxy for differential exposure to the reform. Vietnam comprises 54 ethnic groups. The Kinh constitutes 85% of the population as the majority and concentrated in urban areas. The other 53 minority groups have lower enrollment rates with heterogeneity within ethnic minority groups (S6 Fig) and are geographically dispersed from the north to the south. Son preference also appears to vary significantly across ethnic groups, ranging from a near-normal sex ratio at birth of around 105 among the Muong to highly skewed ratios of 111–115 among the Kinh and Thai [16]. According to the Census 1989, the share of students having completed primary education is lower (S7 Fig), and the share of never-attended students is higher in the ethnic minority group (S8 Fig) than the ethnic majority group due to remoteness and language barriers [17,18]. The compulsory education law aims to improve access in remote and disadvantaged areas (Article 6) and offer tuition-free primary education (Article 13). Thus, by design, it is expected to have a larger impact on these poorer ethnic minority groups living in geographically segregated areas.

**Fig 2:**
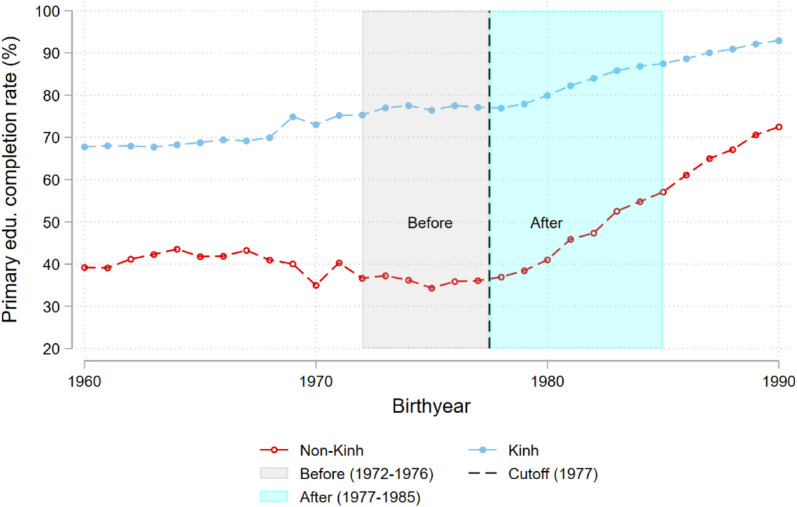
Primary education completion rate. Notes: Fig 2 shows changes in primary education completion rates by ethnicity from 1960 to 1990.

**Fig 3:**
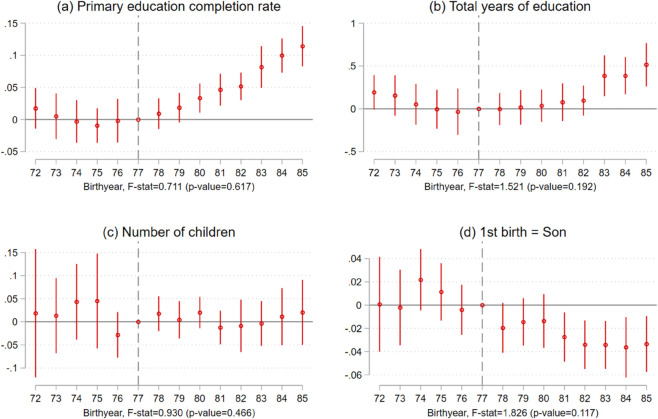
Event-study estimates for education and fertility outcomes. Notes: Fig 3 displays event-study results for primary education completion rate, total years of education, the total number of children, and a binary taking 1 if the first child is son. The base year is 1977.

Fig 2 provides descriptive support for the parallel trends between the two groups. Importantly, the gap in primary education completion rate between the Kinh and the Non-Kinh is stable before 1977, and there was a catch-up by the Non-Kinh after 1977. The critical assumption for the causal identification here is that the gap between the Kinh and the Non-Kinh would remain unchanged in the absence of compulsory education. Though the parallel trends for the post periods cannot be directly tested, Fig 2 shows that the incremental improvement in primary education completion rate emerged right after 1977 and the corresponding event study implies that the parallel trends assumption is not violated (Panel (a) of Fig 3). Panels (b)-(d) of Fig 3 display the estimates from event-study regressions for the total years of education, fertility at the intensive margin and son-starting behavior, respectively. The joint hypotheses tests for the parallel trends for the pre-periods are not rejected. I run the same event-study regressions for the outcome analyzed in the rest of papers and display the results in S9 Fig. The obtained results suggest that the key assumption holds. Nevertheless, the observed statistical insignificance could be driven simply by the lack of statistical power or the selection biases from the conventional pre-trends test [19]. In Sect 6, with the smoothness restriction, I conduct a sensitivity analysis by allowing for potential violations of the parallel trends assumption.

The impact of compulsory education on birth-order-specific son preference is conceptually ambiguous, as it likely influences parents’ preferences for gender and the number of children simultaneously. The literature using the Demographic Health Surveys (DHS) suggests that maternal education is negatively associated with son preference. Raza (2023) [20], for instance, shows that increase in the level of education reduces the proportion of the women who prefer sons over daughters. Similarly but more robustly, Nguyen & Le (2022) [21] explore between-biological-sibling variations in 69 developing countries and find the negative association. While education reduces preference for sons through labor participation, access to information, and assortative matching [21–23], it might strengthen the desire to have a son at the first order due to a declining total fertility rate. As shown in Fig 1, son-starting behavior is more pronounced in households with a smaller number of children. A body of literature report that education decreases fertility by an increase in opportunity costs of leaving school and labor market [15,24], a delay in the onset of marriage and childbearing [24–27], and the shift of preference from quantity to quality [25,28]. If this is the case, households would have fewer chances to try again to achieve their desired number of sons and then son-starting-behavior might increase.

### 3.2 Estimation strategy

To estimate the effects of compulsory education on son-starting behavior, I run the following baseline regression:

$$Y_{ieja}=\beta_{0}+\beta_{1}(Non-Kinh_{\text{e}}\times After_{\text{j}})+\delta_{e}+\lambda_{j}+{𝐗}_{i}^{\prime }\gamma +\theta_{a}+\varepsilon_{ieja}$$ (1)

where *Y*_*ieja*_ refers to education and fertility outcome variables of woman *i* with ethnicity *e* born in year *j* living in area *a*. $Non\;-\;Kinh_{\text{e}}$ is an indicator variable taking 1 if woman *i* belongs to ethnic minority *e*. $\delta_{e}$ is ethnicity fixed effect, capturing any time-invariant systematic differences across ethnic groups(Kinh (base), Hmong, Tay, Thai, Muong, and other minority groups). $After_{\text{j}}$ is an indicator variable taking 1 if woman *i* is born in 1977 or after. $\lambda_{j}$ is cohort fixed effects, absorbing any variations due the timing of birth including the binary of ${\text{After}}_{j}$. $X_{i}$ is a vector of religion controls including Buddhist, Christian, others and no religion (base). $\theta_{a}$ represents area fixed effects, defined by 63 provinces and urban status (i.e., 126 areas in total) based on the current residence. Standard errors are clustered at the ethnicity and birth cohort level. $\beta_{1}$ is the coefficient of interest, representing the intention-to-treat effects of compulsory education on the outcome variables, rather than the average treatment effects of the treated, as the estimate conceptually captures exposure to the reform rather than the actual take-up.

## 4 Main results

Table 1 presents difference-in-differences estimates regarding the effects of compulsory primary education from Eq 1. Column (1) and Column (2) show that compulsory education increases the literacy rate by 6.52 percentage points and the primary education completion rate by 5.69 percentage points. Although it significantly reduces the likelihood of completing secondary school(Column (3)), in total, the years of education increases by 0.14 (Column (4)).

**Table 1:** Main results.

	(1)	(2)	(3)	(4)	(5)	(6)	(7)
	Literacy	Primary	Secondary	Edu.	At Least	# of	First Birth
		Edu.	Edu.	Years	One Child.	Child.	= Son
Non-Kinh × After	0.0652***	0.0569***	-0.0516***	0.1375**	0.0411***	–0.0108	–0.0313***
	(0.0105)	(0.0094)	(0.0092)	(0.0601)	(0.0094)	(0.0229)	(0.0057)
Ethnicity FEs	Yes	Yes	Yes	Yes	Yes	Yes	Yes
Cohort FEs	Yes	Yes	Yes	Yes	Yes	Yes	Yes
Religion Controls	Yes	Yes	Yes	Yes	Yes	Yes	Yes
Area FEs	Yes	Yes	Yes	Yes	Yes	Yes	Yes
Mean of Dep. Var.	0.9351	0.7286	0.3132	8.8069	0.8382	2.0605	0.5488
N	693,960	693,960	693,960	693,960	693,960	581,709	581,709
Adjusted R-squared	0.2560	0.2140	0.2224	0.3218	0.0594	0.1164	0.0025

Note: The sample universe is women born between 1972 and 1985. Standard errors clustered at the birth year and ethnicity level are in parentheses; *, **, and *** denote significance at the 10%, 5%, and 1% levels, respectively.

Exposure to the education policy has positive effects on fertility at the extensive but null effects at the intensive margins (Columns (5) & (6)). Note that these positive effects were not driven by differential treatment under the two-child policy in 1988, as all cohorts were arguably equally affected by the fertility control measure at their reproductive ages. Column (7) presents the results about the relationship between compulsory education and the likelihood of having a son at the first birth order. The negative coefficient indicates that increased exposure to compulsory education mitigates son-starting behavior. Given that the mean of son-starting behavior among ethnic minority women born before 1977 is 0.61, the estimated reduction of 0.03 represents an economically meaningful effect: a decline of approximately 5.09 percent ($\frac{0.03}{0.61}\approx 0.0509$). The full results displaying the coefficients of ethnic fixed effects are available in S5 Table.

The event-study plots in Fig 2 and S9 Fig suggest that the policy had a larger effect on women who were more exposed to it. To examine this further, I divide the treatment group into two cohorts: those born between 1977 and 1979, and those born between 1980 and 1985. S6 Table presents the results. As expected, the magnitude of the effects is larger for the cohort with greater exposure to the policy.

If the reduction in son-starting behavior among ethnic minority women is driven solely by those with only one child, then conceptually, it becomes difficult to distinguish son-starting behavior from son-stopping behavior. To investigate this, I stratify the sample by the number of children and estimate the same specification within each subgroup. The results should be interpreted with caution, as conditioning on the number of children may introduce bias since fertility is both an outcome of the policy reform and interdependent with gender selection. Columns (1)–(3) of S7 Table show that the negative associations are consistently observed not only in women with one child, but also two and three children.

## 5 Potential mechanisms

From the policy perspective, understanding how the beneficial effect of education is mediated matters but the literature is scarce. Nguyen & Le (2022) [21] exploit variations within biological siblings in 69 developing countries by using the DHS and find that education is negatively correlated with son preference. Their analysis on the mechanism suggests that the beneficial effect is mediated through labor participation in non-agriculture market, improvement in access to information and assortative matching. This paper adds on their finding from the two distinctive perspectives. First, the focuses of this paper is the causal relationship between education and son preference. The second is about measurement of son preference. Nguyen & Le (2022) construct son preference based on the ideal number of boys and girls answered by women at the reproductive age but the measurement might suffer from social desirability biases or biases from the past fertility records or future fertility potential. This paper, in contrast, focuses on the gender of the first-born child born to women who have arguably completed their family planning. With this note, I employ the same specification as Eq 1 to assess relationships with a set of labor, marriage and husbands’ education outcomes.

Table 2 reports the results of labor market and marriage outcomes. The compulsory primary education increases the likelihood of obtaining a job in the agricultural sector (Column (1)). This is not exactly consistent with Nguyen & Le (2022) [21] who report the number of educational years completed is positively associated with non-agriculture employment. There is no effect on women’s mobility, defined as a binary indicator equal to 1 if a woman migrated across provinces within the five years preceding the survey (Column (2)). As Anderson & Eswaran (2009) [29] suggest, the critical factor for female empowerment through labor market may be the extent to which employment enhances women’s control over resources. Since the Census data lacks variables capturing this aspect, further research using alternative datasets is necessary to explore the labor market pathways in greater detail.

**Table 2:** Labor market and marriage outcomes.

	(1)	(2)	(3)
	Agri. work	Migrate	Ever married
Non-Kinh × After	0.0132**	0.0002	0.0071***
	(0.0065)	(0.0011)	(0.0019)
Ethnicity FEs	Yes	Yes	Yes
Cohort FEs	Yes	Yes	Yes
Religion Controls	Yes	Yes	Yes
Area FEs	Yes	Yes	Yes
Mean of Dep. Var.	0.3504	0.0221	0.9399
N	598,614	693,960	693,960
Adjusted R-squared	0.3107	0.0503	0.0324

Notes: The sample universe is women born between 1972 and 1985. Standard errors clustered at the birth year and ethnicity level are in parentheses; *, **, and *** denote significance at the 10%, 5%, and 1% levels, respectively.

The link between compulsory education and son-starting behavior may be mediated through marriage market. The parental patterns, bargaining power between parents, and quality of husbands could be changed if female with more education is harnessed to find a partner with more gender-neutral preference. Columns (3) of Table 2 implies that compulsory education increase the likelihood of having ever married by 0.71 percentage points. Furthermore, Columns (1)-(3) of Table 3 show that the treated women are likely to marry men with higher human capital.

**Table 3:** Husbands’ human capital.

	(1)	(2)	(3)
	Literacy	Primary	Edu.
		Edu.	Years
Non-Kinh × After	0.0611***	0.0718***	0.3579***
	(0.0079)	(0.0102)	(0.0583)
Ethnicity FEs	Yes	Yes	Yes
Cohort FEs	Yes	Yes	Yes
Religion Controls	Yes	Yes	Yes
Area FEs	Yes	Yes	Yes
Mean of Dep. Var.	0.9542	0.7515	8.9266
N	552,789	552,789	552,789
Adjusted R-squared	0.1427	0.1729	0.2748

Notes: The sample universe is married women born between 1972 and 1985. Standard errors clustered at the birth year and ethnicity level are in parentheses; *, **, and *** denote significance at the 10%, 5%, and 1% levels, respectively.

There could be other potential paths such as reduced patrilocality and less demand for sons as a substitute of social security [30]. If primary education increases employment that improves actual possession of income [29], it could contribute to greater autonomy and potentially more influence over fertility decisions. However, these are not testable given the data available, and are thus suggested as areas for future research.

## 6 Robustness checks

I conduct a series of robustness checks, and the underlying interpretation of the main results reported in Table 1 remains unchanged.

### 6.1 The 2003 population ordinance

A potential concern in my research design is whether variations stemming from ethnicity and birth year proxy factors other than the education reform and systematically confounds the outcomes. In relation to son-starting behavior, the implementation of the 2003 Population Ordinance and the the improvement in accessibility to sex-selective technologies would be of great concern if those two factors differentially affected ethnic groups and before and after the 1991 threshold of the education reform. The Ordinance explicitly outlaws the identification of fetuses and sex selection. If the ethnic line and the cutoff birth year of 1977 coincidentally capture variations in the sex ratio at the first birth order due to the Ordinance, then it becomes empirically challenging to attribute the observed changes in son-starting behavior between ethnic minority groups to the compulsory education reform in 1991.

Though this is not directly testable as the information on neither the compliance to the Ordinance nor the frequency of abortion is available, I indirectly assess this story in two ways. First, S10 Fig plots the sex ratio at birth of the first children by birth year of “children” with 10-year window periods from the implementation of the Ordinance. Regardless of the birth timing, the ratio started to decrease for both ethnic groups from the 1990s, converging to the same level after the 2000s. Noticeably, this conversion to the gender neutral state started from a way before 2003 and is less likely to stem from 2003, implying that the Ordinance was not the trigger of the decline. Another way is to run a regression of Eq 1 only with women giving the first birth before 2003 (S8 Table). The obtained result suggests that a decline in son-starting behavior is likely driven by those who were born before 2003, the children not affected by the Ordinance.

### 6.2 Expansion of sex-selective technologies

Another challenge is the expansion of sex-selective technologies. While, to the best of the author’s knowledge, the Vietnamese government does not publish official statistics about the number of abortions induced by sex-selective technologies, the literature documents that urltrasound rapidly spread since the 2000s at the affordable cost with heterogeneous expansions between urban and rural areas [31]. According to Jung (2023) [3] who uses the Population Change and Family Planning Survey, the share of mothers who used ultrasound scans for prenatal sex identification rose around 80% in 2005 to 90% in 2013 for urban areas and around 65% in 2005 to 85% in rural areas. As ethnic minorities have a higher son preference and concentrate more on rural areas, this disproportionate improvement is expected to exacerbate son-starting behavior after the 2000s. In the context of S10 Fig, there would be a noticeable increase in the probability of having a son as the first child after the 2000s. No such systematic jumps for the ethnic minority groups imply that the effect of expansion is less likely a leading factor that drove variations in son-starting behavior. To further verity the potential effects of the expansion of the technologies, I include region-specific trends by leveraging the descriptive fact that there exists pronounced heterogeneity in the sex ratio at birth across eight regions. Though this does not completely absorb the variations generated through the expansion as the current regional residency may not correspond to the location of women’s birth place, the main result remains unchanged (S9 Table), giving additional support to the research design.

### 6.3 Deceased children and sample selection

As discussed in Sect 2, women who had at least one deceased child were excluded to accurately determine the birth order of children. However, if the death of female children, in particular, is positively associated with son preference, excluding these samples could lead to an underestimation (overestimation) of the law’s effect on son-starting behavior. This would be the case if the law’s effect were stronger (weaker) for the women who were excluded. Though insignificant, the negative effect of the law on the likelihood of having at least one deceased child, as shown in S10 Table, suggests that removing these women could confound the estimates. To assess the potential impact, I imputed the gender of the first birth for women who had at least one deceased child, using the information on the gender of the deceased children. As shown in S11 Table, the negative effect on son-starting behavior remains.

### 6.4 Two-child policy and the secondary education law

Although the country experienced war between 1965 and 1975, in contrast to the Biafran War in Nigeria [32], the conflict was not fought along ethnic lines. The government introduced a two-child policy in 1988, from which ethnic minorities were exempt [33]. This could bias the estimates if it altered the total (completed) number of children between ethnic minority and majority groups around the 1977 threshold. However, since teenage births constitute only a small share of total fertility—only 0.03 percent of the children were born to the women in the analytical sample before 1988—this is unlikely to be a major concern. Therefore, the ethnic-specific effects of fertility control can be considered similar shock regardless of birth year, and thus are likely captured as a time-invariant ethnic characteristic. A similar rationale applies to the compulsory secondary education law enacted in 2005, which does not affect all birth cohorts in the sample.

### 6.5 Other robustness checks

First, following Rambachan & Roth (2019) [19], I conduct a sensitivity analysis by allowing for potential violations of the parallel-trends assumption. Given that the outcomes are observed in a relatively long periods from the time of primary education to the time of childbearing, I assume that potential confounding factors stem from the existence of secular trends between the ethnic majority and minority groups, rather than shocks that differentially affect the two groups. S11 Fig displays each post-period estimate under the smoothness restrictions. Though the statistical significance disappears for non-linear violations of parallel trends (*M* > 0), the main findings hold when differential linear trends exist (*M* = 0) for all of the outcomes but son-starting behavior. Second, I exclude the year of 1977 and reduce the window size to three years. As shown in S12 Table, the direction of effects and the statistical significance are consistent with the results in Table 1.

## 7 Discussions and conclusions

I analyze the evolution of birth-order-specific son preference and the effect of compulsory primary education on the male-skewed preference using four waves of Vietnam’s census data. The findings reveal that son-starting behavior has increased over time, particularly in households with one or two children. Difference-in-differences estimates suggest that increased women’s education can reduce son-starting behavior. A mechanism analysis implies that this favorable effect may be mediated through a higher likelihood of marrying more educated husbands.

One limitation of this research is that actual exposure to, or compliance with, the compulsory education reform is not observed and needs to be indirectly inferred from women’s ethnic status and birth year. As such, the difference-in-differences estimates should be interpreted as intention-to-treat effects rather than average treatment effects of the treated. In this regard, the beneficial effect of the policy on son-starting behavior is driven by improvement in educational opportunities for girls, not necessarily by the mother’s own education. Relatedly, it is empirically challenging to estimate the local average treatment effect of education on fertility outcomes, since many events—such as migration, employment, and marriage—can occur between the time of schooling and childbearing. This presents a fundamental challenge in finding an instrument that plausibly satisfies the exclusion restriction.

Another limitation of this study is the challenge of disentangling the role of husbands. Fertility decisions result from intra-household bargaining [34], making it difficult to determine whether the observed decline in son-preferring behavior is primarily driven by improvements in women’s education or by their husbands’ education. Using a sample of men, I find similar effects, as shown in S13 Table, suggesting that husbands’ education may also play a role. To assess whether the presence of husbands is a sufficient condition for the observed effect, I restrict the sample to single mothers. The results in S14 Table indicate that the beneficial impact of the education reform on reducing son-starting behavior persists even among women without husbands. Although empirically challenging, identifying plausibly exogenous variation in husband-specific characteristics, conditional on marriage and pre-childbearing status remains an important task for future research.

Despite these limitations, the findings of this study underscore the importance of considering birth order when analyzing son preference, and they highlight another favorable dimension of expanding educational access. Whether Vietnam’s sex ratio at birth will eventually normalize, as seen in countries like China, India, and South Korea, depends on whether shifts in distorted gender preferences can outpace the fertility decline associated with rising opportunity costs of childbearing. This will be an important avenue for future study.

## Supporting information

S1 Table Sample characteristics of women at 35 or above in 1989. (PDF)

S2 Table Sample characteristics of women at 35 or above in 1999. (PDF)

S3 Table Sample characteristics of women at 35 or above in 2009. (PDF)

S4 Table Sample characteristics of women at 35 or above in 2019. (PDF)

S5 Table Main results (full). (PDF)

S6 Table Main results by exposure. (PDF)

S7 Table Other fertility outcomes. (PDF)

S8 Table Robustness check on the 2003 Ordinance. (PDF)

S9 Table Robustness check on the expansion of sex-detective technologies. (PDF)

S10 Table Child mortality. (PDF)

S11 Table Robustness check by imputing the gender of deceased children. (PDF)

S12 Table Robustness check by using a smaller window. (PDF)

S13 Table Main results with the sample of men. (PDF)

S14 Table Main results with the sample of single mothers. (PDF)

S1 FigSex composition and birth order from 1989 to 2019, conditional on mother’s age is 40 or above.S1 Fig displays a simple descriptive statistics about the sex composition of siblings given the total number of siblings for mothers at age 40 or above. The gender-neutral state is represented by $52.5=50\;\times \;\frac{105}{100}$ The statistics are weighted by sampling weights.(TIFF)

S2 FigSex composition and birth order from 1989 to 2019, conditional on mother’s age is 45 or above.S2 Fig displays a simple descriptive statistics about the sex composition of siblings given the total number of siblings for mothers at age 45 or above. The gender-neutral state is represented by $52.5=50\;\times \;\frac{105}{100}$ The statistics are weighted by sampling weights.(TIFF)

S3 FigSex composition and birth order from 1989 to 2019, conditional on mother’s ethnicity is Kinh.S3 Fig displays a simple descriptive statistics about the sex composition of siblings given the total number of siblings for mothers from ethnic majority group. The gender-neutral state is represented by $52.5=50\;\times \;\frac{105}{100}$ The statistics are weighted by sampling weights.(TIFF)

S4 FigSex composition and birth order from 1989 to 2019, conditional on mother’s ethnicity is Non-Kinh.S4 Fig displays a simple descriptive statistics about the sex composition of siblings given the total number of siblings for mothers from ethnic minority groups. The gender-neutral state is represented by $52.5=50\;\times \;\frac{105}{100}$ The statistics are weighted by sampling weights.(TIFF)

S5 Fig Schooling delay in the 1980s. The Census 1989 is used for calculation.
(TIFF)

S6 Fig Primary education completion rate by ethnicity. (TIFF)

S7 Fig Primary education completion rate in the 1980s. The Census 1989 is used for calculation.
(TIFF)

S8 Fig Share of those have never attended school. The Census 1989 is used for calculation.
(TIFF)

S9 Fig Event-study regressions. (TIFF)

S10 Fig Distribution of son-starting behavior 10 years before/after the 2003 Ordinance. (TIFF)

S11 FigSensitivity analysis for the potential violations of parallel trends assumption.S11 Fig plots the event-study estimates with the 95% confidence interval with the Honest DiD bounds suggested by Rambachan and Roth (2019) [19]. The lighter shaded region corresponds to non-linear violations, while the darker region represents linear violations for specific values of *M* for each outcome. Taking the same approach as Dustman et al.(2022) [35], the median of average (absolute) deviations from the trend in the pre-policy period is chosen as the upperbound of *M* for each outcome. M={0.01,0.01,0.02,0.12,0.02,004,0.02} for literacy, primary education, secondary education, years of education, having at least one child, the number of children, and son-starting behavior.(TIFF)
