# Supplementary material for: The evolution of birth-order-specific son preference and compulsory primary education: Evidence from Vietnam
Source: PLoS One. 2025 Dec 1;20(12):e0335527. doi: 10.1371/journal.pone.0335527 (PMC12668500; doi:10.1371/journal.pone.0335527)
Supplement: S2 Table — (PDF) [file pone.0335527.s002.pdf]

S2 Table. Sample characteristics of women at 35 or above in 1999.

	All	With biological mom	No deceased child	No multiples	No discrepancy
	(1)	(2)	(3)	(4)	(5)
	Mean	Mean	Mean	Mean	Mean
Child					
Female	0.47	0.47	0.47	0.47	0.48
Age	13.77	13.76	13.76	13.76	13.10
Number of Siblings	4.15	4.04	3.87	3.86	3.49
Child's Mother					
Ethnicity (Kinh)	0.85	0.85	0.86	0.86	0.86
Christian	0.08	0.08	0.07	0.07	0.07
Buddhist	0.11	0.11	0.11	0.11	0.10
Other Religion	0.02	0.02	0.02	0.02	0.01
Literacy	0.90	0.90	0.92	0.92	0.93
Primary Edu. or Higher	0.58	0.58	0.61	0.61	0.65
Secondary Edu. or Higher	0.15	0.15	0.16	0.16	0.18
Years of Education	6.37	6.37	6.62	6.62	6.86
Married	1.00	1.00	1.00	1.00	1.00
Non-Agricultural Job	0.36	0.36	0.38	0.38	0.40
Child's Household					
Urban	0.46	0.46	0.48	0.48	0.50
Two-Parents	0.68	0.68	0.68	0.68	0.70
Single-Parent	0.08	0.08	0.09	0.09	0.09
Extended	0.07	0.07	0.07	0.07	0.07
N	568,205	565,809	492,782	487,511	370,192

Notes:S2 Table shows how the observable characteristics of children change at each screening step: (i) whether children live with their biological mother (Column (2)), (ii) whether children have at least one deceased sibling (Column (3)), (iii) whether children have twins or triplets as siblings, and (iv) whether the total number of siblings matches the number of surviving children reported by their mothers.
