# Supplementary material for: The evolution of birth-order-specific son preference and compulsory primary education: Evidence from Vietnam
Source: PLoS One. 2025 Dec 1;20(12):e0335527. doi: 10.1371/journal.pone.0335527 (PMC12668500; doi:10.1371/journal.pone.0335527)
Supplement: S5 Table — (PDF) [file pone.0335527.s005.pdf]

S5 Table. Main results (full).

	(1)<br>Literacy	(2)<br>Primary<br>Edu.	(3)<br>Secondary<br>Edu.	(4)<br>Edu.<br>Years	(5)<br>At Least<br>One Child.	(6)<br># of<br>Child.	(7)<br>First Birth<br>= Son
Non-Kinh × After	0.0652***	0.0569***	-0.0516***	0.1375**	0.0411***	-0.0108	-0.0313***
	(0.0105)	(0.0094)	(0.0092)	(0.0601)	(0.0094)	(0.0229)	(0.0057)
Others	-0.2735***	-0.4027***	-0.1229***	-3.6487***	-0.0593***	0.2202***	0.0233***
	(0.0077)	(0.0088)	(0.0073)	(0.0612)	(0.0108)	(0.0252)	(0.0065)
Tay	-0.0625***	-0.1049***	0.0256*	-0.5315***	-0.0952***	-0.0124	0.0225**
	(0.0112)	(0.0133)	(0.0138)	(0.0986)	(0.0177)	(0.0286)	(0.0086)
Thai	-0.2156***	-0.4260***	-0.1876***	-3.7902***	-0.0581***	-0.1424***	0.0458***
	(0.0181)	(0.0244)	(0.0119)	(0.1940)	(0.0104)	(0.0247)	(0.0092)
Muong	-0.0659***	-0.1970***	-0.1220***	-1.7782***	-0.0695***	-0.1098***	0.0264**
	(0.0100)	(0.0146)	(0.0110)	(0.0965)	(0.0125)	(0.0284)	(0.0110)
Hmong	-0.7173***	-0.6504***	-0.2202***	-7.0564***	-0.0108	1.1323***	0.0448***
	(0.0226)	(0.0226)	(0.0222)	(0.2399)	(0.0155)	(0.0270)	(0.0106)
Cohort FEs	Yes	Yes	Yes	Yes	Yes	Yes	Yes
Religion Controls	Yes	Yes	Yes	Yes	Yes	Yes	Yes
Area FEs	Yes	Yes	Yes	Yes	Yes	Yes	Yes
Mean of Dep. Var.	0.9351	0.7286	0.3132	8.8069	0.8382	2.0605	0.5488
N	693,960	693,960	693,960	693,960	693,960	581,709	581,709
Adjusted R-squared	0.2560	0.2140	0.2224	0.3218	0.0594	0.1164	0.0025

Note: The sample universe is women born between 1972 and 1985. Standard errors clustered at the birth year and ethnicity level are in parentheses; *, **, and *** denote significance at the 10%, 5%, and 1% levels, respectively.
