# Supplementary material for: The evolution of birth-order-specific son preference and compulsory primary education: Evidence from Vietnam
Source: PLoS One. 2025 Dec 1;20(12):e0335527. doi: 10.1371/journal.pone.0335527 (PMC12668500; doi:10.1371/journal.pone.0335527)
Supplement: S7 Table — (PDF) [file pone.0335527.s007.pdf]

S7 Table. Other fertility outcomes.

	First Birth = Son			Last Birth = Son		
	(1)<br># of Chid.<br>= 1	(2)<br># of Chid.<br>= 2	(3)<br># of Chid.<br>= 3	(4)<br># of Chid.<br>= 1	(5)<br># of Chid.<br>= 2	(6)<br># of Chid.<br>= 3
Non-Kinh × After	-0.0381***	-0.0215***	-0.0507***	-0.0381***	-0.0343***	-0.0287**
	(0.0078)	(0.0054)	(0.0108)	(0.0078)	(0.0057)	(0.0119)
Ethnicity FEs	Yes	Yes	Yes	Yes	Yes	Yes
Cohort FEs	Yes	Yes	Yes	Yes	Yes	Yes
Religion Controls	Yes	Yes	Yes	Yes	Yes	Yes
Area FEs	Yes	Yes	Yes	Yes	Yes	Yes
Mean of Dep. Var.	0.5870	0.5639	0.4714	0.5870	0.5639	0.4689
N	116,910	344,378	95,754	116,910	344,378	95,754
Adjusted R-squared	0.0097	0.0051	0.0053	0.0097	0.0060	0.0055

Notes: The sample universe is women born between 1972 and 1985. Standard errors clustered at the birth year and ethnicity level are in parentheses; *, **, and *** denote significance at the 10%, 5%, and 1% levels, respectively.
