# Supplementary material for: The evolution of birth-order-specific son preference and compulsory primary education: Evidence from Vietnam
Source: PLoS One. 2025 Dec 1;20(12):e0335527. doi: 10.1371/journal.pone.0335527 (PMC12668500; doi:10.1371/journal.pone.0335527)
Supplement: S8 Table — (PDF) [file pone.0335527.s008.pdf]

S8 Table. Robustness check on the 2003 Ordinance.

	(1) Born before 2003	(2) Born in or after 2003
Non-Kinh × After	-0.0203**	0.0072*
	(0.0086)	(0.0042)
Ethnicity FEs	Yes	Yes
Cohort FEs	Yes	Yes
Religion Controls	Yes	Yes
Area FEs	Yes	Yes
Mean of Dep. Var.	0.5945	0.5334
N	146,134	435,575
Adjusted R-squared	0.0100	0.0004

Notes: The sample universe is women born between 1972 and 1985. Standard errors clustered at the birth year and ethnicity level are in parentheses; *, **, and *** denote significance at the 10%, 5%, and 1% levels, respectively.
