# Supplementary material for: The evolution of birth-order-specific son preference and compulsory primary education: Evidence from Vietnam
Source: PLoS One. 2025 Dec 1;20(12):e0335527. doi: 10.1371/journal.pone.0335527 (PMC12668500; doi:10.1371/journal.pone.0335527)
Supplement: S9 Table — (PDF) [file pone.0335527.s009.pdf]

S9 Table. Robustness check on the expansion of sex-detective technologies.

	(1) Literacy	(2) Primary Edu.	(3) Secondary Edu.	(4) Edu. Years	(5) At Least One Child.	(6) # of Child.	(7) First Birth = Son
Non-Kinh × After	0.0537***	0.0486***	-0.0471***	0.0871	0.0022	-0.0495***	-0.0177***
	(0.0112)	(0.0110)	(0.0098)	(0.0777)	(0.0069)	(0.0182)	(0.0058)
Ethnicity FEs	Yes	Yes	Yes	Yes	Yes	Yes	Yes
Cohort FEs	Yes	Yes	Yes	Yes	Yes	Yes	Yes
Religion Controls	Yes	Yes	Yes	Yes	Yes	Yes	Yes
Area FEs	Yes	Yes	Yes	Yes	Yes	Yes	Yes
Region-specific Trends	Yes	Yes	Yes	Yes	Yes	Yes	Yes
Mean of Dep. Var.	0.9351	0.7286	0.3132	8.8069	0.8382	2.0605	0.5488
N	693,960	693,960	693,960	693,960	693,960	581,709	581,709
Adjusted R-squared	0.2567	0.2145	0.2231	0.3221	0.0631	0.1196	0.0028

Notes: The sample universe is women born between 1972 and 1985. Standard errors clustered at the birth year and ethnicity level are in parentheses; *, **, and *** denote significance at the 10%, 5%, and 1% levels, respectively.
