# Supplementary material for: The evolution of birth-order-specific son preference and compulsory primary education: Evidence from Vietnam
Source: PLoS One. 2025 Dec 1;20(12):e0335527. doi: 10.1371/journal.pone.0335527 (PMC12668500; doi:10.1371/journal.pone.0335527)
Supplement: S10 Table — (PDF) [file pone.0335527.s010.pdf]

S10 Table. Child mortality.

	Had at Least One Deceased Child		
	(1)	(2)	(3)
	Overall	Son	Daughter
Non-Kinh × After	-0.0009	-0.0003	-0.0011
	(0.0013)	(0.0012)	(0.0008)
Ethnicity FEs	Yes	Yes	Yes
Cohort FEs	Yes	Yes	Yes
Religion Controls	Yes	Yes	Yes
Area FEs	Yes	Yes	Yes
Mean of Dep. Var.	0.0165	0.0106	0.0077
N	591,467	591,467	591,467
Adjusted R-squared	0.0051	0.0039	0.0020

Notes: The sample universe is women born between 1972 and 1985. Standard errors clustered at the birth year and ethnicity level are in parentheses; *, **, and *** denote significance at the 10%, 5%, and 1% levels, respectively.
