# Supplementary material for: The evolution of birth-order-specific son preference and compulsory primary education: Evidence from Vietnam
Source: PLoS One. 2025 Dec 1;20(12):e0335527. doi: 10.1371/journal.pone.0335527 (PMC12668500; doi:10.1371/journal.pone.0335527)
Supplement: S11 Table — (PDF) [file pone.0335527.s011.pdf]

S11 Table. Robustness check by imputing the gender of deceased children.

	(1)<br>First Birth<br>= Son
Non-Kinh × After	-0.0313***
	(0.0057)
Ethnicity FEs	Yes
Cohort FEs	Yes
Religion Controls	Yes
Area FEs	Yes
Mean of Dep. Var.	0.5504
N	591,467
Adjusted R-squared	0.0026

Notes: The sample universe is women born between 1972 and 1985. For the gender of the deceased children, if all of the deceased children reported are female(male), the gender of the first birth is imputed as female(male). If women had both male and female deceased children, the gender of the first birth is imputed as male. Standard errors clustered at the birth year and ethnicity level are in parentheses; *, **, and *** denote significance at the 10%, 5%, and 1% levels, respectively.
