# Supplementary material for: The evolution of birth-order-specific son preference and compulsory primary education: Evidence from Vietnam
Source: PLoS One. 2025 Dec 1;20(12):e0335527. doi: 10.1371/journal.pone.0335527 (PMC12668500; doi:10.1371/journal.pone.0335527)
Supplement: S12 Table — (PDF) [file pone.0335527.s012.pdf]

S12 Table. Robustness check by using a smaller window.

	(1) Literacy	(2) Primary Edu.	(3) Secondary Edu.	(4) Edu. Years	(5) At Least One Child.	(6) # of Child.	(7) First Birth = Son
Non-Kinh × After	0.0229***	0.0247***	-0.0268***	0.0110	0.0283***	-0.0050	-0.0242***
	(0.0054)	(0.0057)	(0.0080)	(0.0416)	(0.0084)	(0.0218)	(0.0052)
Ethnicity FEs	Yes	Yes	Yes	Yes	Yes	Yes	Yes
Cohort FEs	Yes	Yes	Yes	Yes	Yes	Yes	Yes
Religion Controls	Yes	Yes	Yes	Yes	Yes	Yes	Yes
Area FEs	Yes	Yes	Yes	Yes	Yes	Yes	Yes
Mean of Dep. Var.	0.9267	0.6914	0.2592	8.2995	0.8308	2.0723	0.5546
N	268,217	268,217	268,217	268,217	268,217	222,841	222,841
Adjusted R-squared	0.2798	0.2135	0.2166	0.3182	0.0456	0.1193	0.0030

Notes: The sample universe is women born between 1974 and 1976 for the non-affected group and between 1978 and 1980 for the affected group. Robust standard errors in parentheses are clustered at the area level. *, **, and *** denote significance at the 10%, 5%, and 1% levels, respectively.
