# Supplementary material for: The evolution of birth-order-specific son preference and compulsory primary education: Evidence from Vietnam
Source: PLoS One. 2025 Dec 1;20(12):e0335527. doi: 10.1371/journal.pone.0335527 (PMC12668500; doi:10.1371/journal.pone.0335527)
Supplement: S13 Table — (PDF) [file pone.0335527.s013.pdf]

S13 Table. Main results with the sample of men.

	(1) Literacy	(2) Primary Edu.	(3) Secondary Edu.	(4) Edu. Years	(5) At Least One Child.	(6) # of Child.	(7) First Birth = Son
Non-Kinh × After	0.0626***	0.1050***	-0.0276***	0.5197***	0.0884***	0.0743***	-0.0267***
	(0.0074)	(0.0119)	(0.0080)	(0.0802)	(0.0092)	(0.0159)	(0.0058)
Ethnicity FEs	Yes	Yes	Yes	Yes	Yes	Yes	Yes
Cohort FEs	Yes	Yes	Yes	Yes	Yes	Yes	Yes
Religion Controls	Yes	Yes	Yes	Yes	Yes	Yes	Yes
Area FEs	Yes	Yes	Yes	Yes	Yes	Yes	Yes
Mean of Dep. Var.	0.9551	0.7665	0.3153	9.1845	0.7894	2.0790	0.5438
N	696,373	696,373	696,373	696,373	696,373	549,706	549,706
Adjusted R-squared	0.0984	0.1595	0.1709	0.2605	0.0706	0.1201	0.0014

Notes: The sample universe is men born between 1972 and 1985. Standard errors clustered at the birth year and ethnicity level are in parentheses; *, **, and *** denote significance at the 10%, 5%, and 1% levels, respectively.
