# Supplementary material for: The evolution of birth-order-specific son preference and compulsory primary education: Evidence from Vietnam
Source: PLoS One. 2025 Dec 1;20(12):e0335527. doi: 10.1371/journal.pone.0335527 (PMC12668500; doi:10.1371/journal.pone.0335527)
Supplement: S14 Table — (PDF) [file pone.0335527.s014.pdf]

S14 Table. Main results with the sample of single mothers.

	(1) Literacy	(2) Primary Edu.	(3) Secondary Edu.	(4) Edu. Years	(5) # of Child.	(6) First Birth = Son
Non-Kinh × After	0.0634***	0.0655***	-0.0568***	0.1702	-0.0241	-0.0407***
	(0.0141)	(0.0118)	(0.0103)	(0.1059)	(0.0243)	(0.0111)
Ethnicity FEs	Yes	Yes	Yes	Yes	Yes	Yes
Cohort FEs	Yes	Yes	Yes	Yes	Yes	Yes
Religion Controls	Yes	Yes	Yes	Yes	Yes	Yes
Area FEs	Yes	Yes	Yes	Yes	Yes	Yes
Mean of Dep. Var.	0.9438	0.7560	0.3994	9.6664	1.6977	0.5366
N	77,599	77,599	77,599	77,599	77,599	77,599
Adjusted R-squared	0.2269	0.1945	0.2417	0.3186	0.0518	0.0018

Notes: The sample consists of single mothers born between 1972 and 1985. Standard errors clustered at the birth year and ethnicity level are in parentheses; *, **, and *** denote significance at the 10%, 5%, and 1% levels, respectively.
