# Supplementary figures and images for: The evolution of birth-order-specific son preference and compulsory primary education: Evidence from Vietnam
Source: PLoS One. 2025 Dec 1;20(12):e0335527. doi: 10.1371/journal.pone.0335527 (PMC12668500; doi:10.1371/journal.pone.0335527)

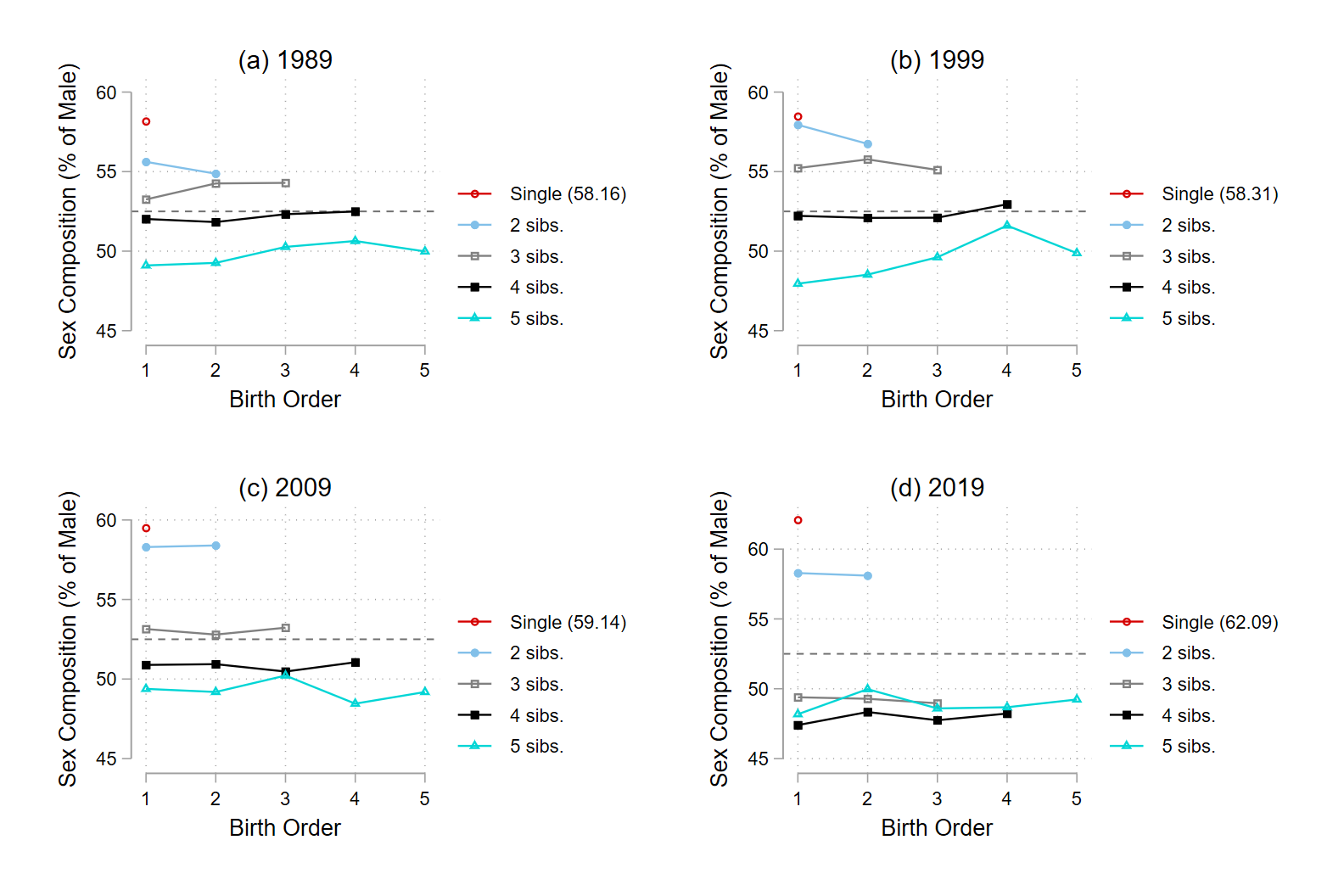

Supplement: S1 Fig — displays a simple descriptive statistics about the sex composition of siblings given the total number of siblings for mothers at age 40 or above. The gender-neutral state is represented by 52.5=50×105100 The statistics are weighted by sampling weights. (TIFF) [file pone.0335527.s015.tif]

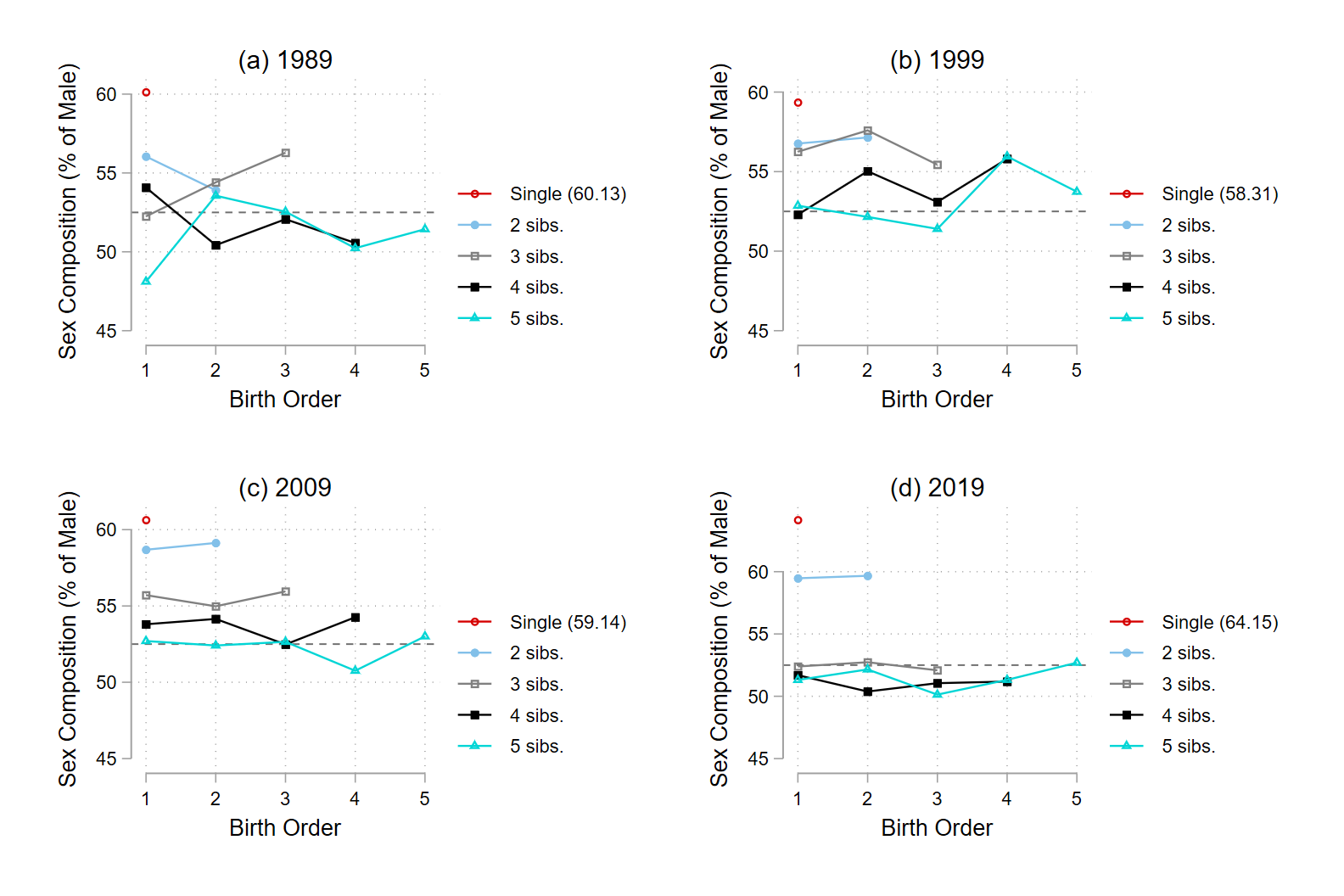

Supplement: S2 Fig — displays a simple descriptive statistics about the sex composition of siblings given the total number of siblings for mothers at age 45 or above. The gender-neutral state is represented by 52.5=50×105100 The statistics are weighted by sampling weights. (TIFF) [file pone.0335527.s016.tif]

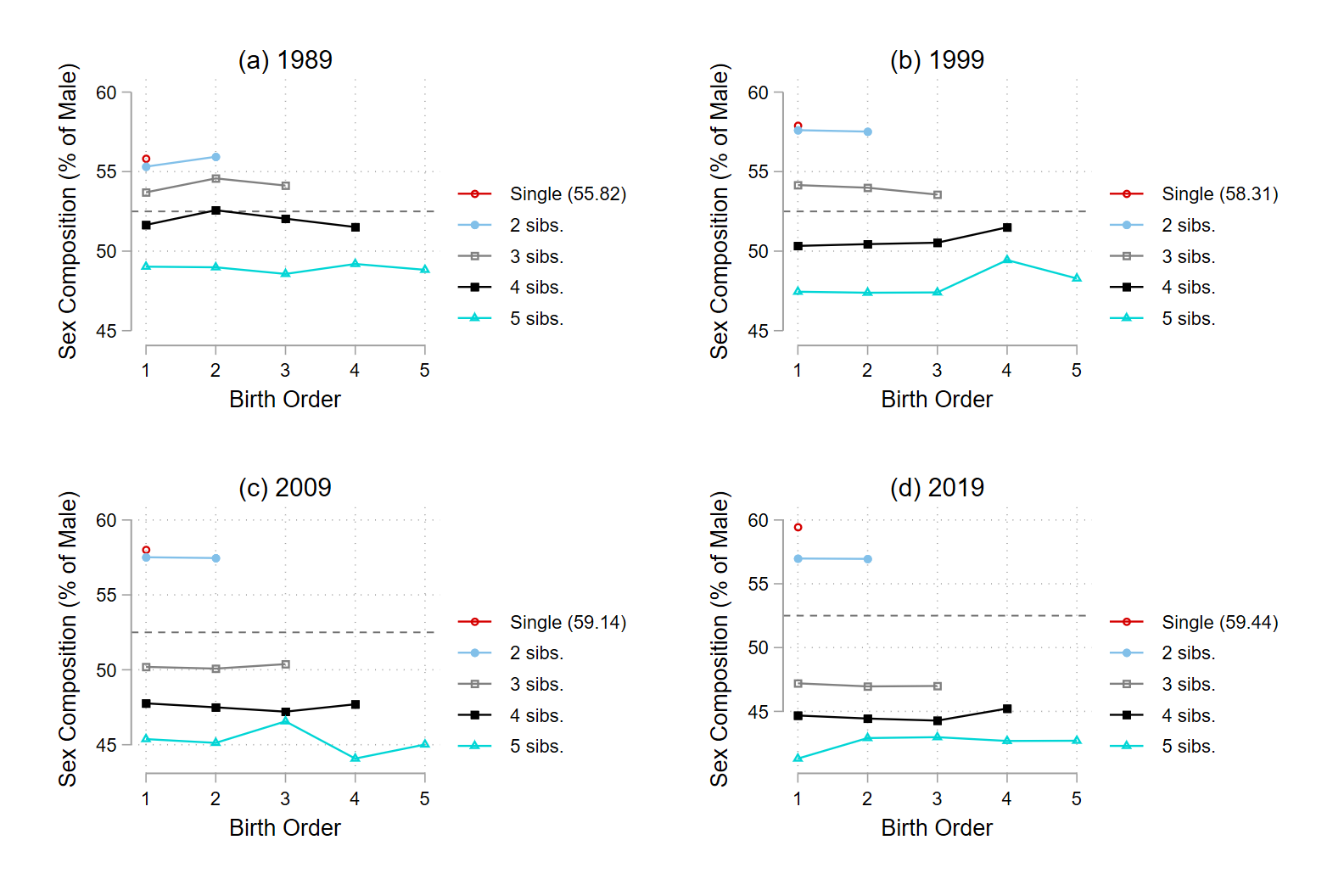

Supplement: S3 Fig — displays a simple descriptive statistics about the sex composition of siblings given the total number of siblings for mothers from ethnic majority group. The gender-neutral state is represented by 52.5=50×105100 The statistics are weighted by sampling weights. (TIFF) [file pone.0335527.s017.tif]

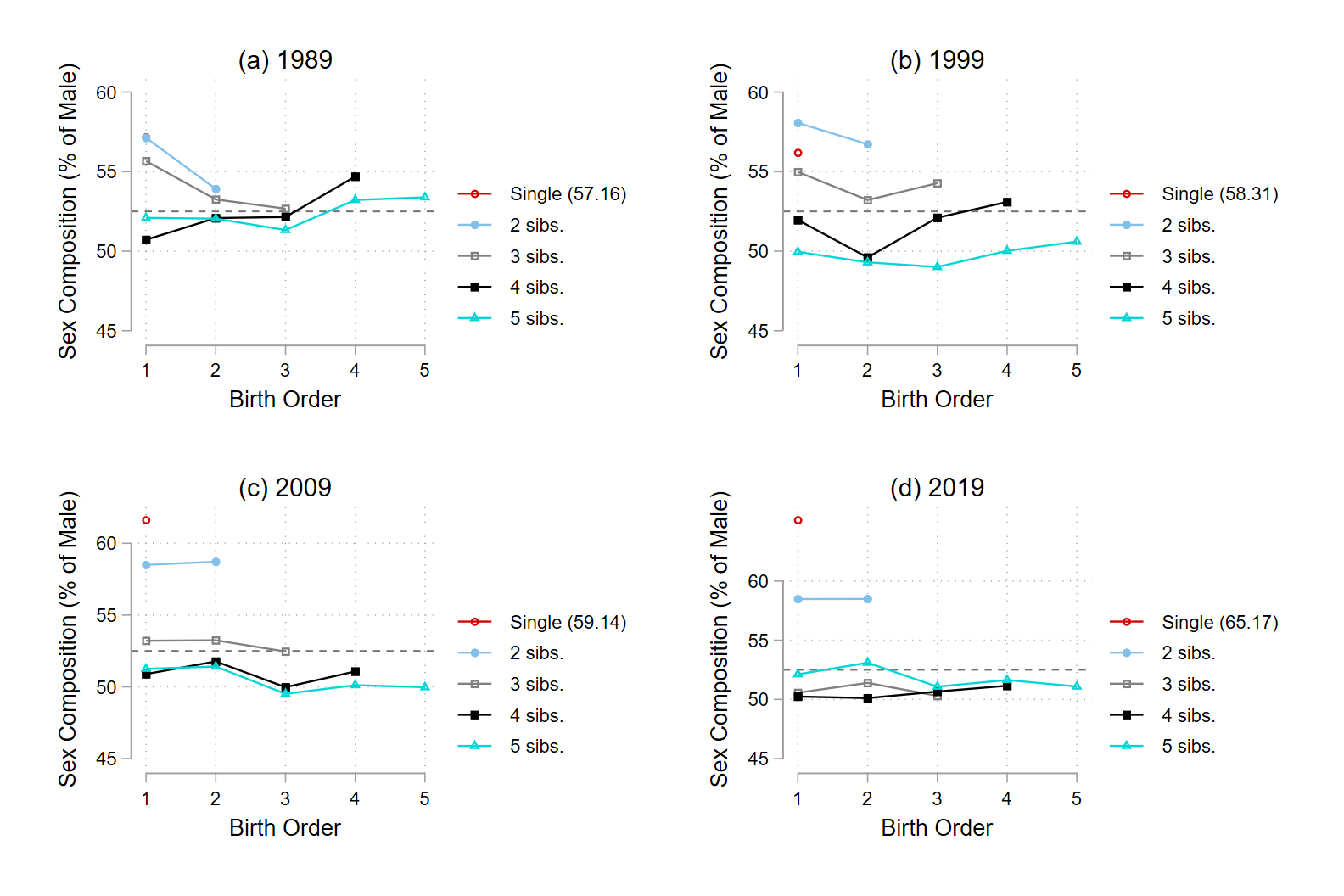

Supplement: S4 Fig — displays a simple descriptive statistics about the sex composition of siblings given the total number of siblings for mothers from ethnic minority groups. The gender-neutral state is represented by 52.5=50×105100 The statistics are weighted by sampling weights. (TIFF) [file pone.0335527.s018.tif]

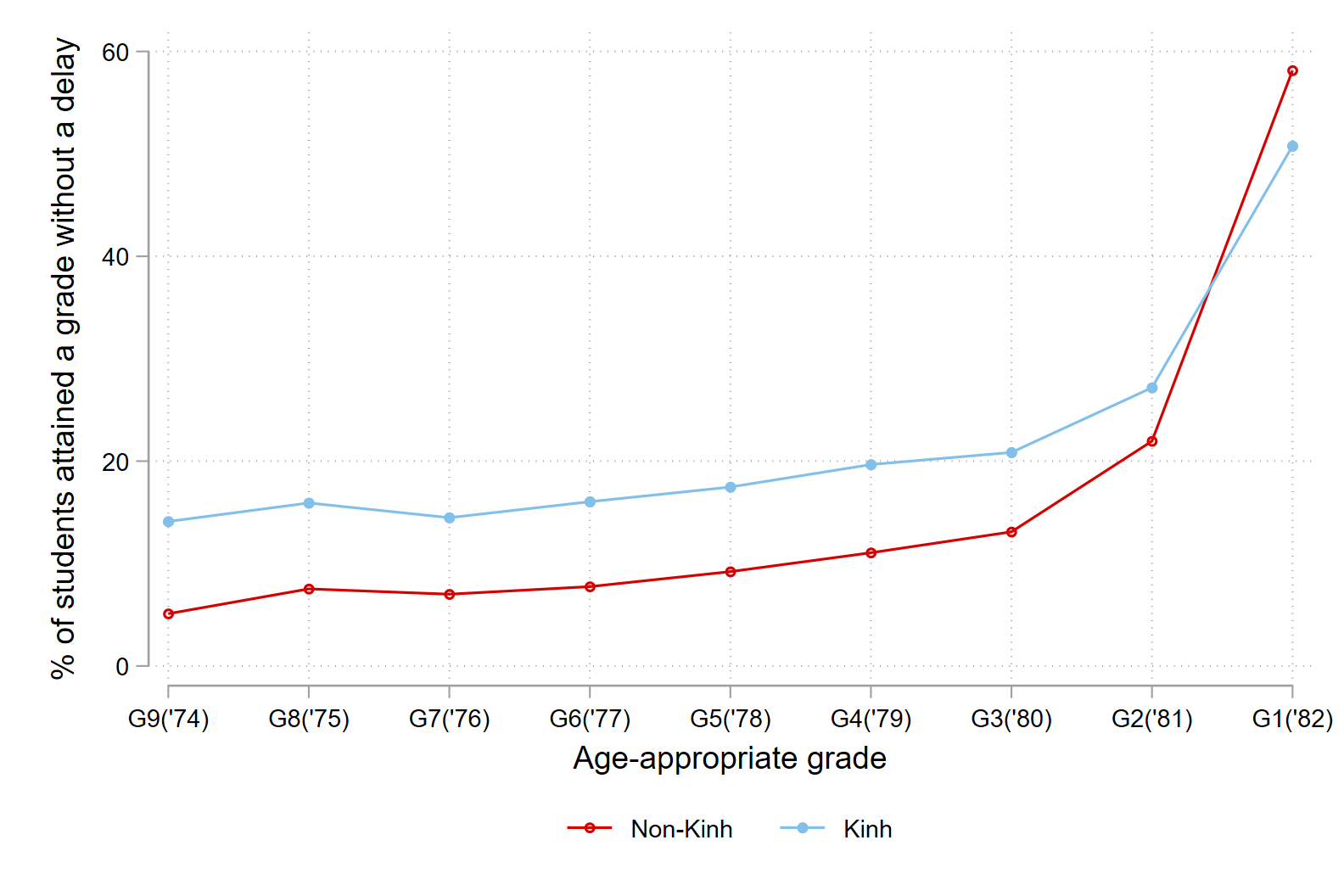

Supplement: S5 Fig — The Census 1989 is used for calculation. (TIFF) [file pone.0335527.s019.tif]

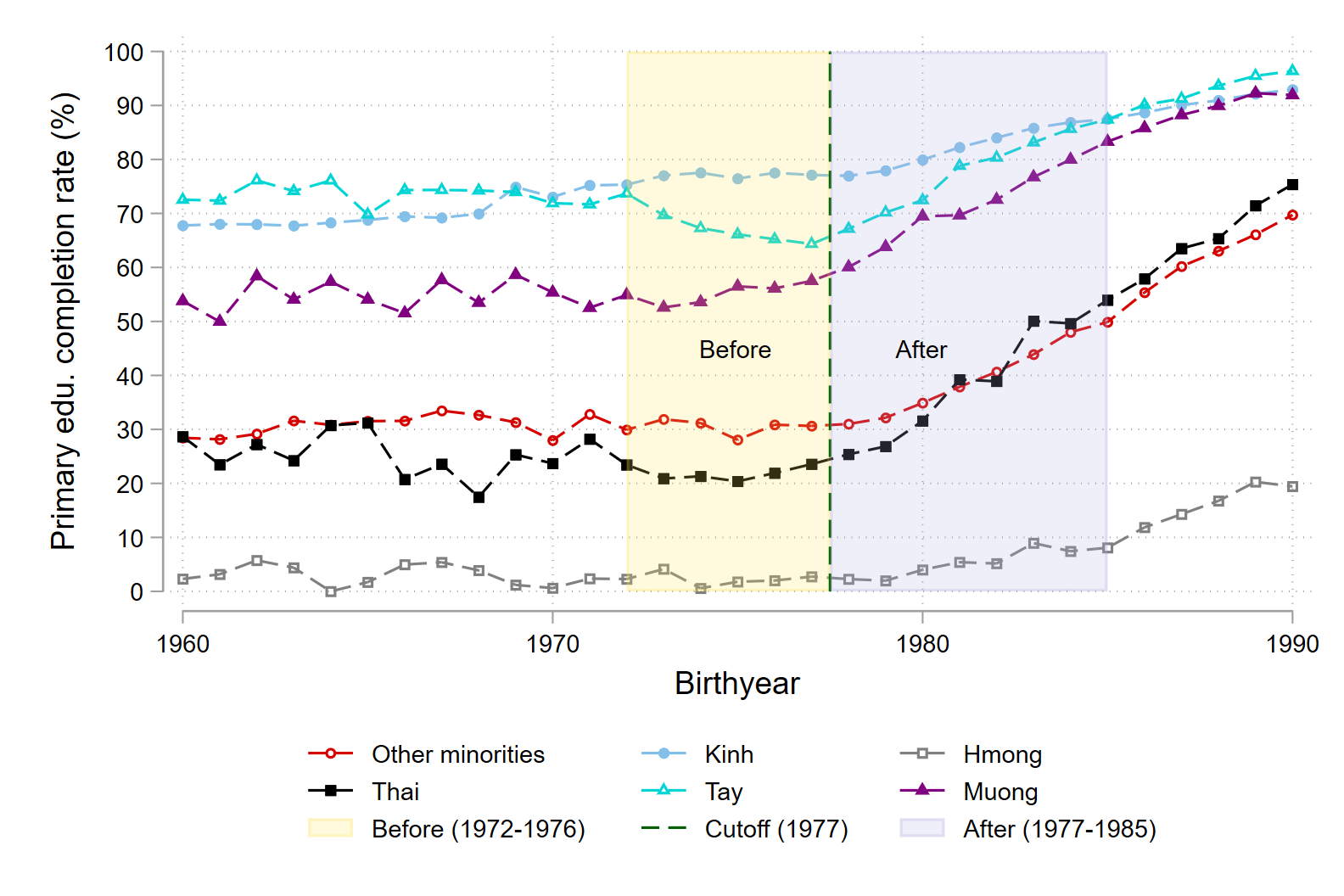

Supplement: S6 Fig — (TIFF) [file pone.0335527.s020.tif]

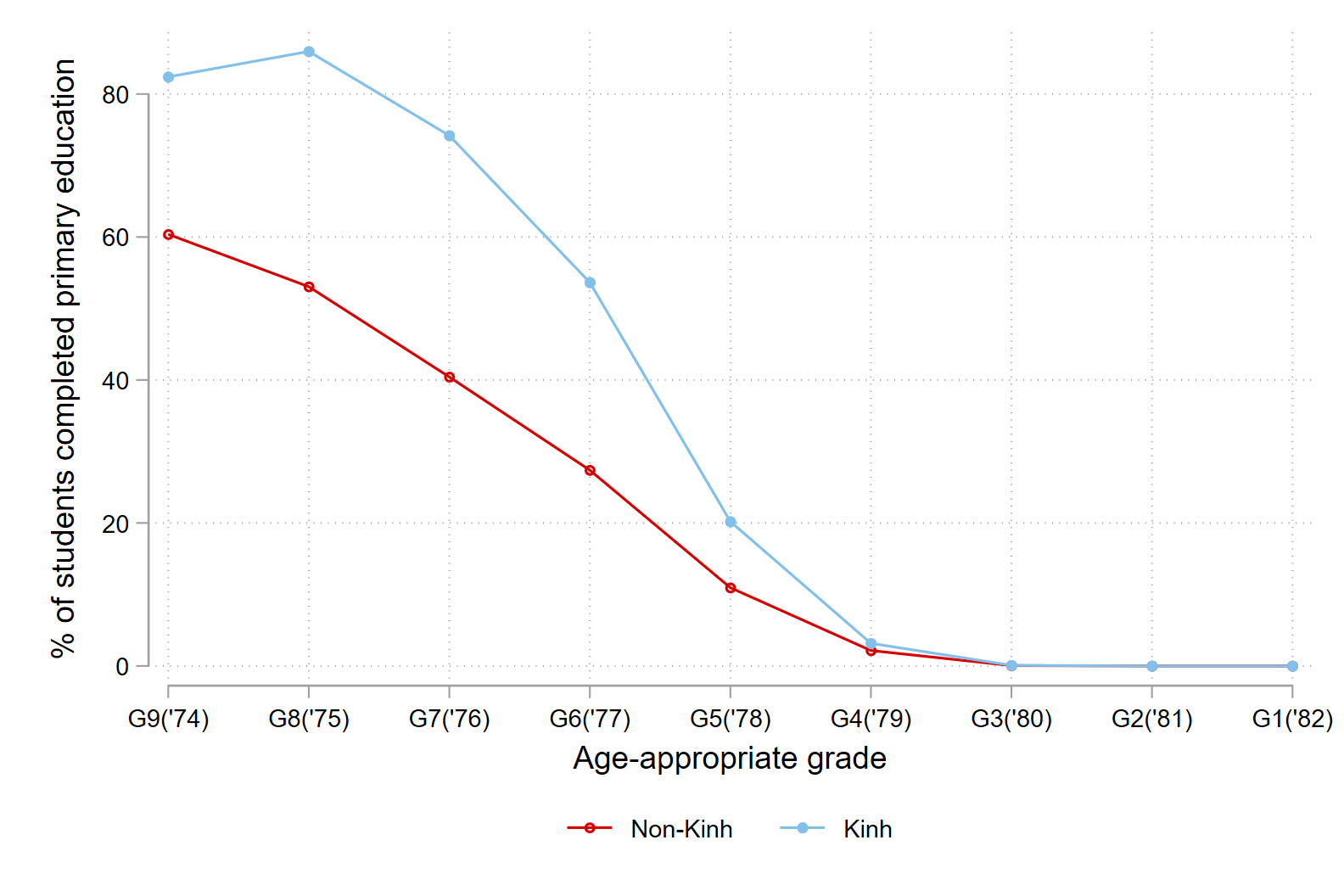

Supplement: S7 Fig — The Census 1989 is used for calculation. (TIFF) [file pone.0335527.s021.tif]

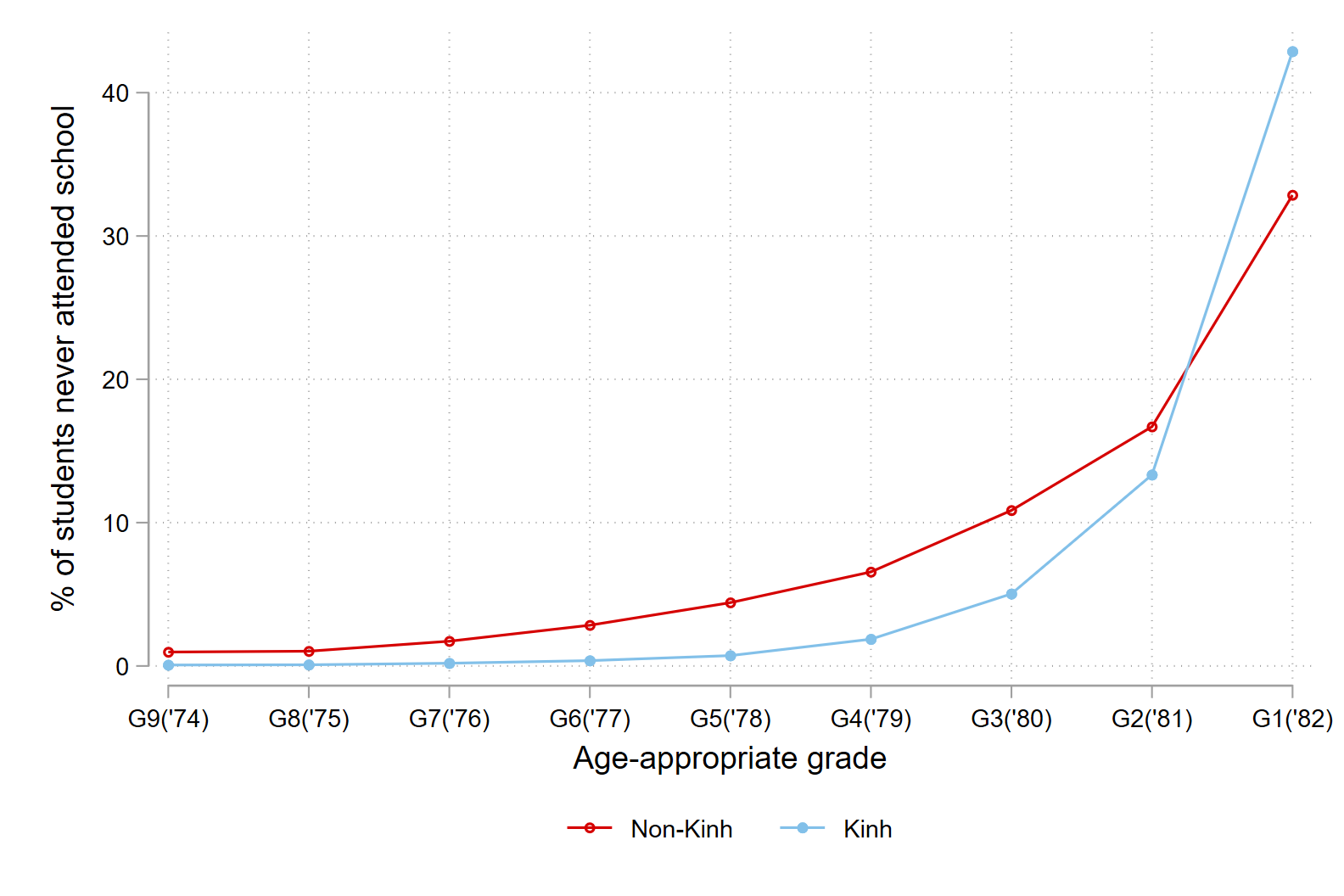

Supplement: S8 Fig — The Census 1989 is used for calculation. (TIFF) [file pone.0335527.s022.tif]

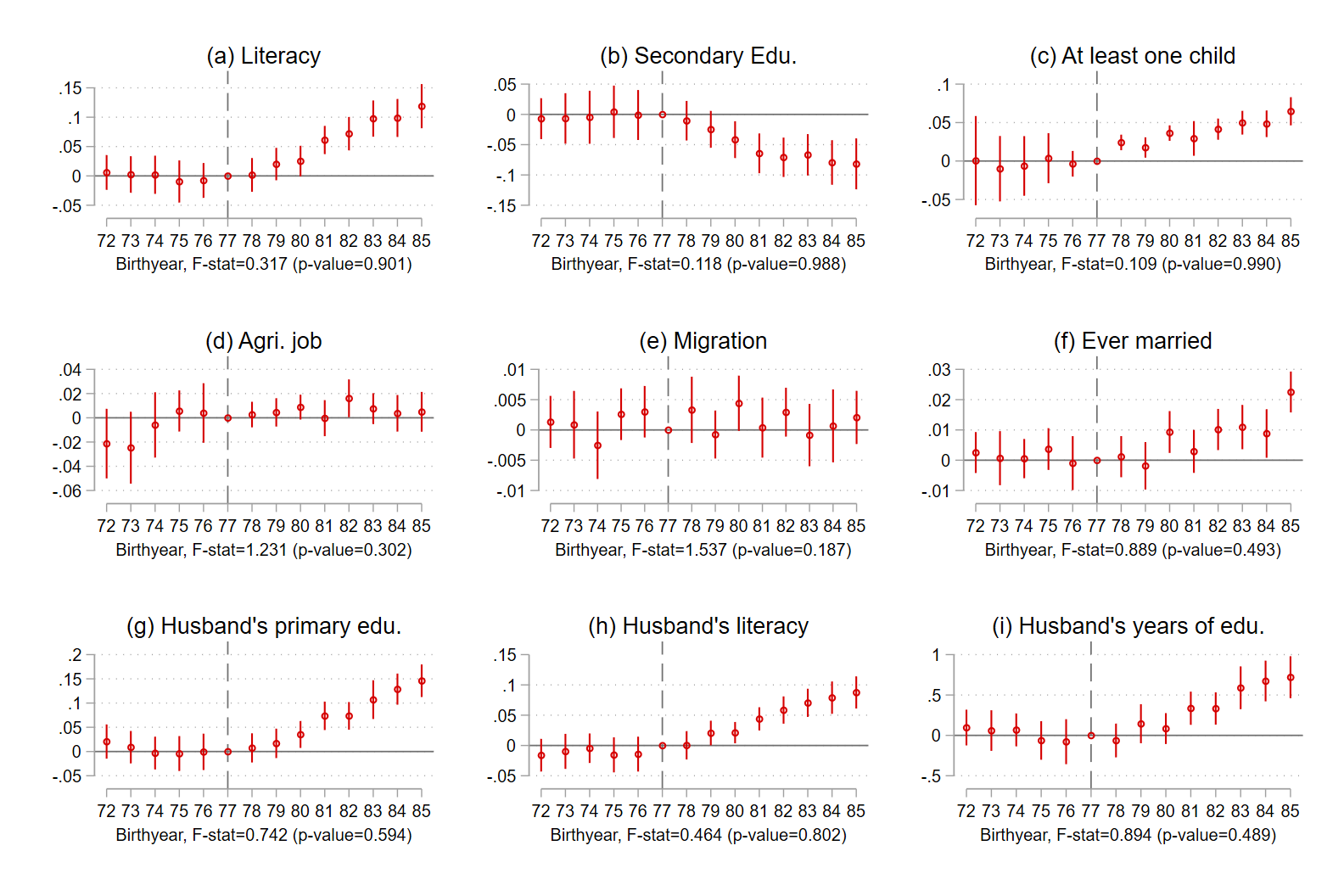

Supplement: S9 Fig — (TIFF) [file pone.0335527.s023.tif]

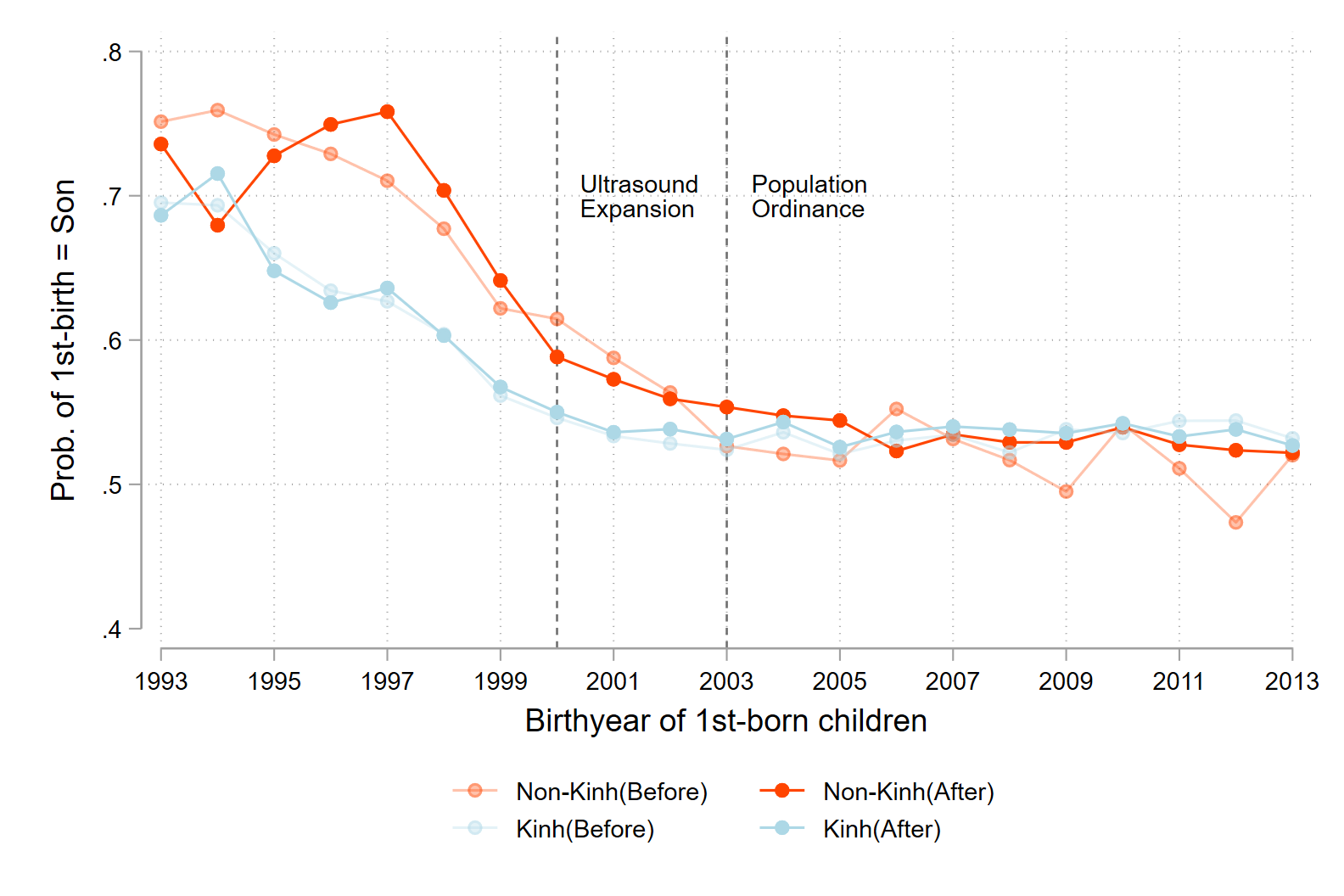

Supplement: S10 Fig — (TIFF) [file pone.0335527.s024.tif]

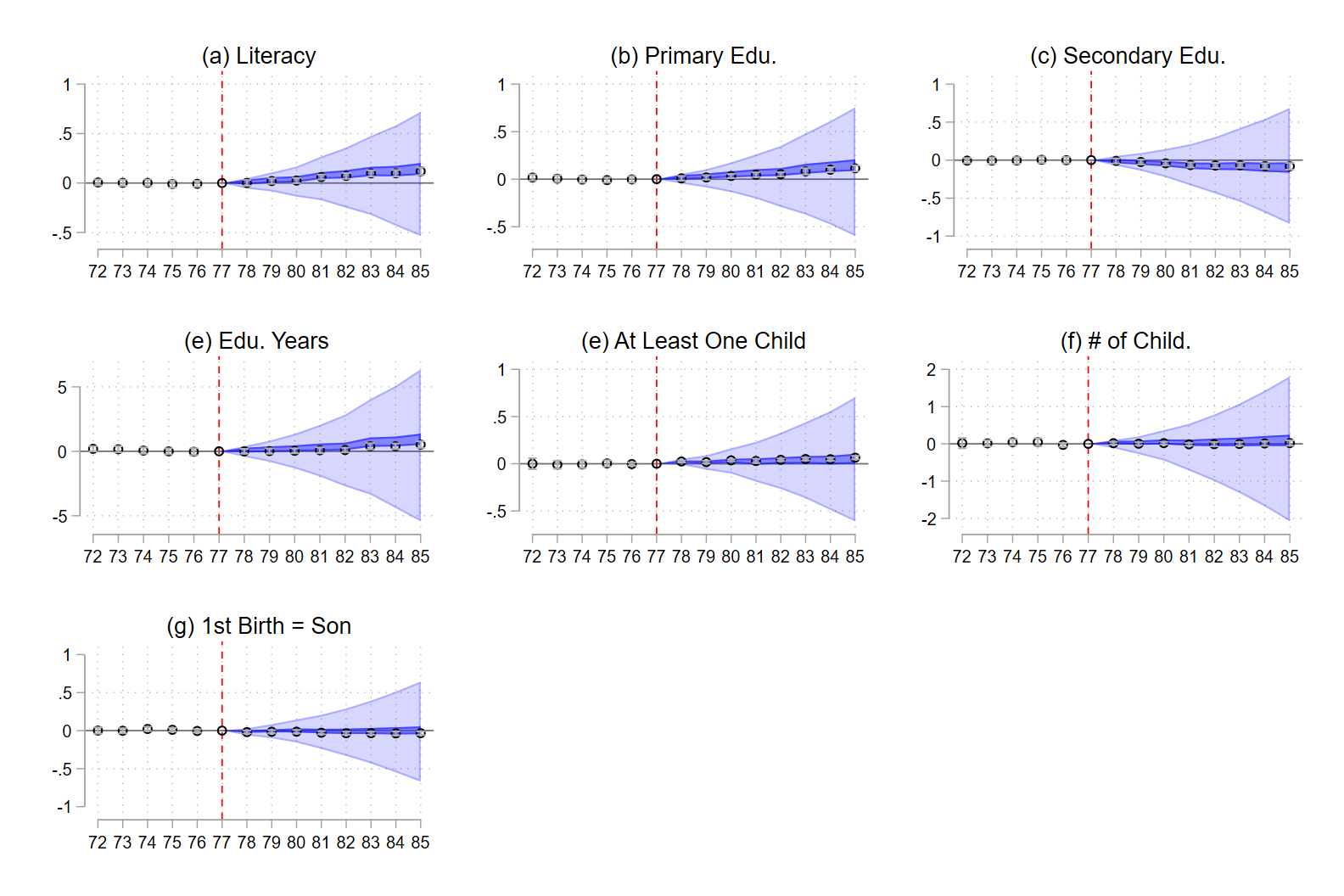

Supplement: S11 Fig — plots the event-study estimates with the 95% confidence interval with the Honest DiD bounds suggested by Rambachan and Roth (2019) [19]. The lighter shaded region corresponds to non-linear violations, while the darker region represents linear violations for specific values of M for each outcome. Taking the same approach as Dustman et al.(2022) [35], the median of average (absolute) deviations from the trend in the pre-policy period is chosen as the upperbound of M for each outcome. M={0.01,0.01,0.02,0.12,0.02,004,0.02} for literacy, primary education, secondary education, years of education, having at least one child, the number of children, and son-starting behavior. (TIFF) [file pone.0335527.s025.tif]
